# Gastric reactive capillary hemangioma caused by tislelizumab

**DOI:** 10.1055/a-2657-0108

**Published:** 2025-08-20

**Authors:** Xin Long, Huipeng Zhang, Tengfei Yin, Jing Zhang, Yanbo Yu, Yanjing Gao

**Affiliations:** 1 91623 Department of Gastroenterology, Qilu Hospital of Shandong University, Jinan, China

A 71-year-old woman was admitted with melena for two months. She was being treated with tislelizumab and oral lenvatinib for hepatocellular carcinoma. Scattered pustular, exfoliative lesions were seen on the lower extremities ( Fig. 1 ). Laboratory tests showed hemoglobin at 49.0 g/L and alpha-fetoprotein at 8073.00 ng/mL. Typically, the cause of melena is esophagogastric variceal bleeding. Contrast-enhanced computed tomography strongly demonstrated gastric malignancy ( Fig. 2 ). Gastroscopy and ultrasound endoscopy revealed a huge cauliflower-like mass in the fundus of stomach and blood flow signals within it ( Fig. 3 , Video 1 ). The pathology of both biopsies showed blood clots and granulation tissue ( Fig. 4 ). As a definitive diagnosis could not be made, the multidisciplinary team discussed the results as a rare programmed death 1 (PD-1)-associated gastric reactive capillary hemangioma and that the lesion was predominantly supplied by the left gastric artery. She then underwent embolization of the left gastric and gastric omental arteries, after which her melena was significantly better. However, she again had an amount of melena four weeks later. She then underwent two endoscopic sclerotherapy sessions, after which the blood flow signal within the lesion disappeared ( Fig. 5 , Video 1 ). At the six-week follow-up, she had no signs of gastrointestinal bleeding.

**Fig. 1:**
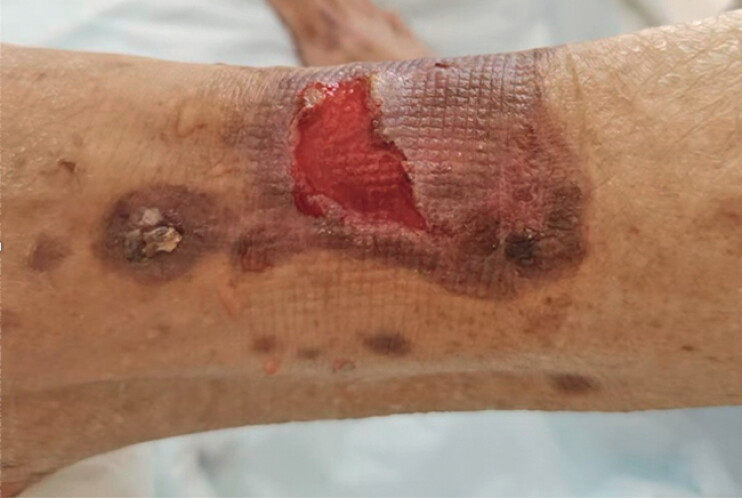
Scattered pustular exfoliative lesions are seen on the lower extremities, partly fused and partly crusted on the surface. Thicker brown scabs are also seen.

**Fig. 2:**
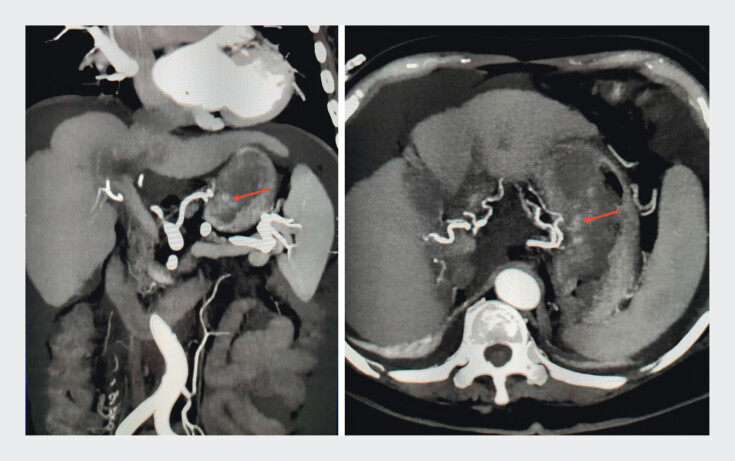
Contrast-enhanced computed tomography (CT) images before endoscopy: gastric wall thickening, uneven and slightly higher density in the lumen, marked enhancement of the lesion on the side of the lesser curvature of the stomach, and abundant blood supply.

**Fig. 3:**
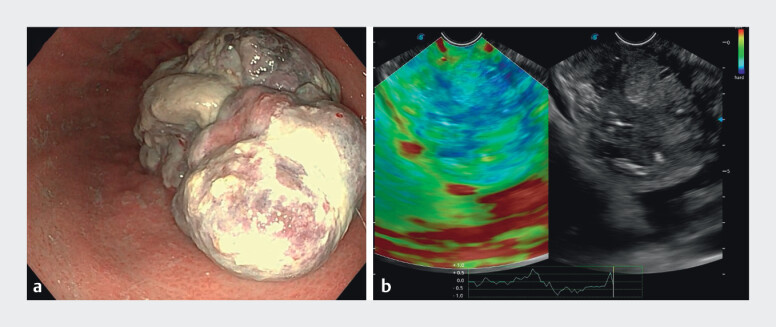
**a** Gastroscopic examination revealed a large, cauliflower-like mass at the gastric base. **b** Endoscopic ultrasonography identified the lesion as a heterogeneously hypoechoic mass with irregular internal echoes, demonstrating intralesional vascularity on color Doppler imaging.

**Fig. 4:**
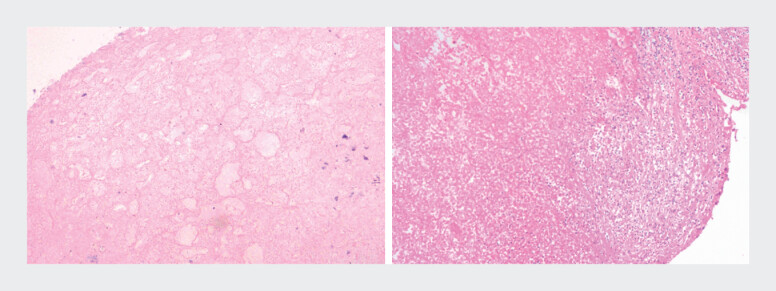
Pathological findings showed blood clots and hemorrhagic necrotic tissue.

**Fig. 5:**
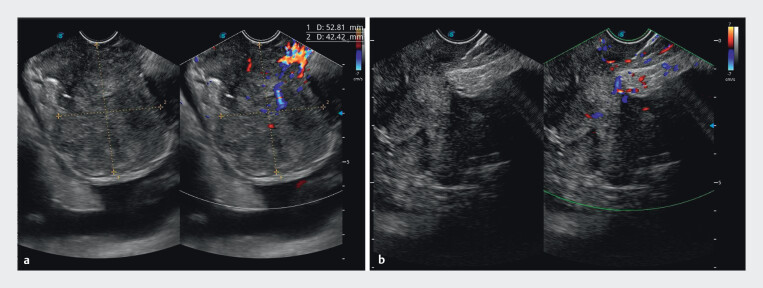
Color doppler flow imaging. **a** Pre-procedural blood flow signal can be seen. **b** The disappearance of the flow signals was observed postoperatively with the application of a COOK 22 G puncture needle for multipoint injection of polidocanol in the lesion vessel.

Use of a COOK 22 G puncture needle for multipoint injection of polidocanol in the lesion vessel.Video 1

Although previous studies have mentioned that reactive capillary hyperplasia occurs mainly in the skin and mucous membranes 1 2 3 , this case suggests that reactive capillary hemangioma may also occur within other deep tissues in PD-1 inhibitor users. However, the pathogenesis, prevention, and therapeutic measures of reactive capillary hemangioma in the gastrointestinal tract require further study.

Endoscopy_UCTN_Code_CCL_1AB_2AD_3AZ

## Notes

Endoscopy E-Videos https://eref.thieme.de/e-videos
*E-Videos* is an open access online section of the journal *Endoscopy* , reporting on interesting cases and new techniques in gastroenterological endoscopy. All papers include a high-quality video and are published with a Creative Commons CC-BY license. Endoscopy E-Videos qualify for HINARI discounts and waivers and eligibility is automatically checked during the submission process. We grant 100% waivers to articles whose corresponding authors are based in Group A countries and 50% waivers to those who are based in Group B countries as classified by Research4Life (see: https://www.research4life.org/access/eligibility/ ). This section has its own submission website at https://mc.manuscriptcentral.com/e-videos .
